# Baisse du HDL-cholestérol indicateur du stress oxydatif dans le diabète de type 2

**DOI:** 10.11604/pamj.2014.19.140.5279

**Published:** 2014-10-13

**Authors:** Arsène Tshikongo Kabamba, Salvius Amuri Bakari, Albert Otshudi Longanga, Zet Kalala Lukumwena

**Affiliations:** 1 Faculté des Sciences Pharmaceutiques, Université de Lubumbashi, République Démocratique Congo; 2 Université Libre de Bruxelles (ULB), Belgique; 3 Faculté de Médecine Vétérinaire, Université de Lubumbashi, République Démocratique Congo

**Keywords:** HDL-Cholestérol, Lubumbashi, Stress oxydatif, Diabète de type 2, HDL-Cholesterol, Lubumbashi, oxydative stress, Type 2 diabetes

## Abstract

L'hypercholestérolémie est étroitement liée au stress oxydatif. Lorsqu'il y a trop de cholestérol qui circule dans le sang, il n'est pas utilisé en totalité par les cellules et il risque de s'accumuler dans les vaisseaux sanguins. Cela peut entrainer la formation des plaques d'athérosclérose qui gênent la circulation sanguine et provoquent des accidents cardiovasculaires. Le stress oxydatif apparait très tôt dans l'histoire des complications du diabète de type 2, et est lié à l'oxydation du glucose mais aussi à la peroxydation lipidique. Le cholestérol-HDL est un marqueur important du stress oxydatif par sa capacité à faciliter la métabolisation du cholestérol, sa baisse est souvent considérée comme la source de beaucoup d'inquiétudes. L'objectif est l’évaluation de la variation du taux de cholestérol-HDL, marqueur du stress oxydatif, chez les patients diabétiques de type 2 dans la population congolaise. Nous avons inclus dans cette étude prospective des cas témoins des patients diabétiques de type 2 reconnus et diagnostiqués, et des témoins non diabétiques appariés selon l’âge et le sexe. Parallèlement au bilan biologique classique, une analyse d'un des facteurs de risque du stress oxydatif a été réalisée: baisse de HDL-Cholestérol. L’âge moyen des 30 patients diabétiques (47,77±10,78 ans) était comparable à celui des 30 témoins (48,83±10,73 ans). Une baisse significative du cholestérol-HDL dans le sang était observée chez 100% des diabétiques et 50% des témoins (p=0,0000). L'augmentation du HDL cholestérol permet d’éliminer le mauvais cholestérol en excès en nettoyant les tissus et en ramenant le cholestérol vers le foie. Lors du diabète de type 2 on constate une baisse sanguine sensible du taux de HDL-cholestérol, qui est signe indicateur du stress oxydatif.

## Introduction

Le HDL-cholestérol est la partie du cholestérol non athérogène. Il peut être abaissé dans de nombreuses circonstances dont l'obésité, la sédentarité, l'hypertension artérielle, le tabagisme, le diabète et l'hyper lipoproteinemie athérogène. Au taux de 60 mg/ dl ou plus, le HDL-cholestérol est considéré comme un marqueur antiathérogène; maintenir le taux élevé de HDL est un objectif régulièrement mentionné dans les recommandations thérapeutiques [1–3]. Dans le diabète de type 2 et dans le syndrome métabolique, il est classique d'observer la présence de petites particules HDL3 dont les propriétés antiathérogènes sont altérées; mais à l'inverse des particules HDL de très grande taille peuvent avoir moins de pouvoir antioxydant ou anti-inflammatoire [4–6]. Le HDL-cholestérol a un rôle athero-protecteur très important; pour chaque augmentation du HDL cholestérol de 10 mg/dl, le risque coronarien diminue de 2% chez l'homme et de 3% chez la femme. Le cholestérol Ester Transfer protein (CETP) est une enzyme qui permet le transfert du cholestérol-HDL vers les adipocytes; son inhibition permet l'augmentation du cholestérol-HDL sanguin et une baisse du LDL-cholestérol [7]. A cause de son insolubilité dans l'eau, le cholestérol nécessite un transporteur (protéine) pour circuler dans le sang. Il existe deux types de lipoprotéines pouvant transporter le cholestérol: les lipoprotéines LDL (Law Density Lipoprotein) qui apportent le cholestérol du foie vers les cellules et les lipoprotéines HDL (High Density Protein) qui récupèrent le cholestérol dans les tissus pour le ramener au foie [8, 9]. Les HDL sont riches en plusieurs protéines antioxydantes comme la paraoxonase et, peuvent prévenir l'oxydation des LDL. De plus, l des effets bénéfiques directs sur l'endothélium vasculaire en stimulant la production du monoxyde d'azote (NO) et en diminuant l'expression des molécules d'adhésion à l'endothélium [10–12]. Les HDL ont également des propriétés anti thrombotiques et anti-infectieuses [12]. C'est la fraction HDL2 qui est responsable des effets bénéfiques, et une sous fraction des HDL ne contenant que l'apolipoprotéine A1 (LpA-1) qui serait la fraction la plus efficace dans la récupération du cholestérol cellulaire. Il y a de nombreuses variations génétiques conduisant certaines personnes à avoir un HDL bas sans majoration du risque oxydatif, et d'autres à risque majoré malgré une valeur haute. Ainsi, un simple taux de HDL est bien loin de pouvoir rendre compte de l'ensemble des propriétés des particules HDL [13]. Une réduction de taille des particules HDL est associée à la résistance à l'insuline et s'accompagne d'une réduction de taille des particules LDL et d'une hypertriglycéridémie [3, 10, 14, 15]. Les relations entre HDL et risque de survenue du stress oxydatif lors du diabète de type 2 sont complexes en raison de l'hétérogénéité des HDL et de leurs activités variées. Ce travail s'inscrit (i) dans la stratégie de prise en charge du stress oxydatif lors du diabète sucré et (ii) dans la prévention des complications du diabète de type 2. Il vise à identifier la variation du cholestérol HDL dans le diabète de type 2.

## Méthodes

Il s'est agi d'une étude prospective réalisée sur une période de 3 mois allant de janvier à Mars 2014. La population étudiée était constituée des adultes diabétiques de type 2 dont le diagnostic a été confirmé par des tests biologiques basés sur la détermination de la glycémie et de l'insulinémie. Les personnes ayant constitué notre échantillon (groupe I) étaient suivis aux cliniques universitaires de Lubumbashi et à l'hôpital Gécamines sud. Ils étaient âgés de 36 à 59 ans sans distinction de sexe. Nous avons pu réunir un total de 30 patients. Chaque cas a été apparié à un témoin. Les personnes ayant constitué le groupe témoin (groupe II) ont été choisies parmi les volontaires avec une glycémie normale.

Les prélèvements ont été effectués à jeun pendant la matinée de 8h00’ à 10h30’ dans les hôpitaux concernés et les échantillons analysés au laboratoire de Biochimie des cliniques universitaires. Les données ont été colligées à partir des dossiers des patients suivis et hospitalisés. Le cholestérol-HDL a été étudiée par la méthode enzymatique. Cette variable a été regroupée selon les valeurs normales de cholestérol dans le sang et les valeurs à risque de cholestérol dans le sang [16, 17].

Les résultats obtenus ont été saisis et analysés sur le logiciel Epi Info 2011 (version 7.0.8.1). Les moyennes sont présentées avec les écarts-types et les Odds ratio (OR) avec un intervalle de confiance à 95% (IC 95%). Le test t de Student a été utilisé pour la comparaison des moyennes et celle des fréquences (exprimées en pourcentage) par le test de X2 corrigé de Yates ou le test exact de Fisher lorsque recommandé. Le seuil de signification a été fixé à p <0,05.

## Résultats

L’âge moyen des 30 patients diabétiques (47,77±10,78 ans) était comparable à celui des 30 témoins (48,83±10,73 ans) (Tableau 1). La moyenne du taux de cholestérol-HDL dans le sang est de 38,9 ± 2 mg/dl dans le groupe I constitué de personnes diabétiques connues. Dans le groupe II, deux tendances ont été observées: chez 50% des personnes la moyenne de cholestérol-HDL a été de 46,3 ± 5 mg/dl alors qu'elle est de 74,3 ± 9,2 mg/dl dans la seconde moitié des individus du groupe II. Le test t de Student donne une différence statistiquement significative en comparant ces moyennes (t=11,21; p=0,0000). En classifiant les résultats du taux de cholestérol-HDL de cette étude en fonction des valeurs normales et des valeurs à risque, il s'avère que 100% de personnes du groupe I présentent un taux à risque du cholestérol-HDL dans le sang contre 50% chez les personnes du groupe II. Chez 50% des personnes du groupe II, la valeur du cholestérol-HDL a été trouvée normale. La comparaison de ces deux proportions donne une différence statistiquement significative (p=0,0000) (Tableau 1).

**Table 1:** Comparaison de l'age et des valeurs de HDL-cholestérol (en mg/dl) des diabétiques de type 2 par rapport aux témoins

Paramètres	Groupe I	(n = 30)	Groupe II	(n = 30)
Age (année)	47,77	±10,78	48,83	± 10,73
HDL-Cholestérol (mg/dl)	38,9	± 2	(n_a_=15)	(n_b_=15)
			46,3 ± 5	74,3 ± 9,2
**Taux à risque de cholestérolémie-HDL**				
	n	%	n	%
<40 mg/dl	15	50	0	0
<50 mg/dl	15	50	3	10
Total	30	100	3	10

*Valeurs de références : Taux normal de HDL-cholestérol dans le sang: 60 mg / dl (1,6 mmol / L) ou au-dessus ; Taux à risque de HDL-cholestérol dans le* sang Homme: Moins de 40 mg / dL (1,0 mmol / L) ; Femme: *Moins de 50 mg / dL (1,3 mmol / L)*

## Discussion

Cette étude a mis en évidence une baisse significative du taux de cholestérol-HDL chez les diabétiques connus de type 2, preuve d'existence du stress oxydatif chez ces individus. La diminution du cholestérol-HDL est directement liée à la baisse du bon cholestérol avec comme conséquences directes la survenue des complications liées à une hypercholestérolémie [4, 18]. Etant donné que lors du diabète, le stress oxydatif est à la base de beaucoup de complications, nous pensons que le taux de HDL-cholestérol peut donner une idée du degré du stress oxydatif. Dans cette étude, la baisse du cholestérol-HDL laisse penser qu'il y a une grande quantité du cholestérol dans le sang qui reste exposer au stress oxydatif ou pouvant induire la dégradation des vaisseaux. Notre observation rejoint celle faite antérieurement par Ben et al. (2009) et ZHAN et al. (2014) [3, 19]. Dans le diagnostic du diabète de type 2, on semble mettre beaucoup d'emphase sur le mauvais profil lipidique à savoir le cholestérol-LDL, les triglycérides et le cholestérol-VLDL. Certes leur augmentation est trop inquiétante [3, 9, 18]; cependant la plupart des mécanismes physiopathologiques du diabète sucré dont le stress oxydatif se traduit sur le plan paraclinique par une diminution importante du cholestérol-HDL [1, 19]; laissant place au mauvais cholestérol: celui-ci pouvant générer par oxydation, des radicaux libres qui sont responsables des atteintes vasculaires comme il est établi que l'oxydation du cholestérol est un élément capital pour la survenue du stress oxydatif [15, 19, 20]. L'idéal pour les diabétiques c'est d'avoir en grande quantité les lipoprotéines HDL qui agissent en ramenant l'excès de cholestérol dans le sang vers le foie au niveau duquel il sera détruit [3, 10, 12, 18]. Ainsi plus le niveau de cholestérol-HDL sera élevé, moins on aura le mauvais cholestérol dans le sang, et moins on aura les complications dues au stress oxydatif.

Cette étude a mis en évidence 50% des personnes à risque dans le groupe témoin. Cette observation laisse penser que ces personnes sont soit des diabétiques qui s'ignorent, soit qu'il s'agit des personnes à risque de le devenir. En effet, la littérature signale qu'en Afrique subsaharienne environs 75% des cas de diabète sucré restent non diagnostiqués [21]. Au vu des observations mentionnées ci-dessus, nous pensons donc d'une part que, l'objectif lors de la prise en charge du diabète de type 2, devra être celui de maintenir normal le taux de cholestérol-HDL pour prévenir le stress oxydatif, et d'autre part, la mesure du cholestérol-HDL pourrait servir d'alarme pour les diabétiques ignorées et les personnes à risques. Chez les diabétiques lorsqu'on constate la baisse de cholestérol-HDL, un traitement anti oxydant devrait être instauré. Les démarches thérapeutiques devraient en même temps se focaliser avec attention sur le cholestérol-HDL comme sur le mauvais cholestérol. Par ailleurs, nous pensons que le fait de juste baisser le taux de cholestérol-LDL peut ne pas être suffisant pour prévenir ou réduire les effets du stress oxydatif chez les personnes diabétiques; mais par contre ces effets pourraient donc être réduits davantage par l'augmentation du taux de cholestérol HDL. Pour autant, bien que des niveaux plus élevés de cholestérol-HDL puissent être utiles pour réduire les risques d'avoir une crise cardiaque, les recherches de CHANG et al. 2013; HIRANO et al. 2010; ARUNA et al. 2014[1, 6, 10] ont soulignés le fait de devoir considérer également d'autres facteurs de risque de développer une maladie cardiaque.

## Conclusion

Dans la présente étude nous avons évalué la variation du taux de cholestérol-HDL chez 60 personnes dont 30 diabétiques de type 2 et 30 individus connus comme non diabétiques à Lubumbashi. L’étude a mis en évidence une diminution sanguine significative de HDL-cholestérol chez les personnes diabétiques de type 2 et chez 50% des personnes auparavant connues comme non diabétiques. Cela est très probablement lié à un degré important traduisant l'oxydation du HDL-cholestérol à la base du stress oxydatif observé chez les sujets ayant un diabète de type 2. L'augmentation de cholestérol-HDL par les antioxydants devra être privilégiée à l'avant plan de traitement du diabète de type 2, car son augmentation permet la baisse du mauvais cholestérol. Les antioxydants peuvent être apportés par l'alimentation. Il est indispensable d'orienter cette approche thérapeutique vers les conseils diététiques pour viser à la fois la diminution du taux de cholestérol-LDL mais aussi l'augmentation du HDL-cholestérol.
